# Cardiovascular and metabolic risk of antipsychotics in children and young adults: a multinational self-controlled case series study

**DOI:** 10.1017/S2045796021000494

**Published:** 2021-10-15

**Authors:** Kenneth K. C. Man, Shih-Chieh Shao, Yu-Chuan Chang, Mei-Hung Chi, Han Eol Jeong, Swu-Jane Lin, Chien-Chou Su, Ju-Young Shin, Kirstie H. Wong, Ian C. K. Wong, Yea-Huei Kao Yang, Yen-Kuang Yang, Edward Chia-Cheng Lai

**Affiliations:** 1 Research Department of Practice and Policy, UCL School of Pharmacy, London, UK; 2 Department of Pharmacology and Pharmacy, Centre for Safe Medication Practice and Research, University of Hong Kong, Hong Kong, People's Republic of China; 3 Laboratory of Data Discovery for Health (D24H), Hong Kong Science Park, Hong Kong, People's Republic of China; 4 School of Pharmacy, Institute of Clinical Pharmacy and Pharmaceutical Sciences, College of Medicine, National Cheng Kung University, Tainan, Taiwan; 5 Department of Pharmacy, Keelung Chang Gung Memorial Hospital, Keelung, Taiwan; 6 Department of Psychiatry, National Cheng Kung University Hospital, Tainan, Taiwan; 7 School of Pharmacy, Sungkyunkwan University, Seoul, South Korea; 8 Department of Pharmacy Systems, Outcomes and Policy, College of Pharmacy, University of Illinois at Chicago, Chicago, IL, USA; 9 Department of Pharmacy, National Cheng Kung University Hospital, Tainan, Taiwan; 10 Department of Paediatrics and Adolescent Medicine, Li Ka Shing Faculty of Medicine, the University of Hong Kong, Hong Kong, People's Republic of China

**Keywords:** Antipsychotics, cardiovascular events, children and young adults, metabolic syndrome, multi-national study, self-controlled case series

## Abstract

**Aims:**

The risk of antipsychotic-associated cardiovascular and metabolic events may differ among countries, and limited real-world evidence has been available comparing the corresponding risks among children and young adults. We, therefore, evaluated the risks of cardiovascular and metabolic events in children and young adults receiving antipsychotics.

**Methods:**

We conducted a multinational self-controlled case series (SCCS) study and included patients aged 6–30 years old who had both exposure to antipsychotics and study outcomes from four nationwide databases of Taiwan (2004–2012), Korea (2010–2016), Hong Kong (2001–2014) and the UK (1997–2016) that covers a total of approximately 100 million individuals. We investigated three antipsychotics exposure windows (i.e., 90 days pre-exposure, 1–30 days, 30–90 days and 90 + days of exposure). The outcomes were cardiovascular events (stroke, ischaemic heart disease and acute myocardial infarction), or metabolic events (hypertension, type 2 diabetes mellitus and dyslipidaemia).

**Results:**

We included a total of 48 515 individuals in the SCCS analysis. We found an increased risk of metabolic events only in the risk window with more than 90-day exposure, with a pooled IRR of 1.29 (95% CI 1.20–1.38). The pooled IRR was 0.98 (0.90–1.06) for 1–30 days and 0.88 (0.76–1.02) for 31–90 days. We found no association in any exposure window for cardiovascular events. The pooled IRR was 1.86 (0.74–4.64) for 1–30 days, 1.35 (0.74–2.47) for 31–90 days and 1.29 (0.98–1.70) for 90 + days.

**Conclusions:**

Long-term exposure to antipsychotics was associated with an increased risk of metabolic events but did not trigger cardiovascular events in children and young adults.

## Introduction

The average prevalence of psychiatric disorders was about 22.1% with the severe disorders (schizophrenia, bipolar disorder, severe depression, severe anxiety and severe post-traumatic stress disorder) estimated to be 5.1% (Charlson *et al*., 2019). In children, the prevalence has been reported to be about 6.7% but varies between different countries (Erskine *et al*., 2017). The use of antipsychotics has increased over the years and has become one of the mainstays for the treatment of psychiatric disorders in children, despite lingering concerns over side effects (Harrison *et al*., 2012; Lao *et al*., 2017; Lee *et al*., 2018). Specifically, in such young populations, antipsychotic medications may induce cardiovascular and metabolic abnormalities (such as obesity, hyperglycaemia, dyslipidaemia and diabetes mellitus) that could affect their physical, mental and social development (Hsu *et al*., 2013). Moreover, some life-threatening cardiovascular side effects of antipsychotics such as stroke, ischaemic heart disease (IHD) and acute myocardial infarction (AMI) have also been reported (De Hert *et al*., 2011). Meanwhile, increasing prescribing of antipsychotics is observed among children and young adults (Olfson *et al*., 2015), which leads to great concern regarding the safety of antipsychotics (Pillay *et al*., 2018). Currently available studies mainly focus on the outcomes of weight change or shifts in metabolic parameters over a short period of time (Sjo *et al*., 2017; Vandenberghe *et al*., 2018). Not much is known about the specific risk of cardiovascular and metabolic events in children and young adults treated with antipsychotics, as previous studies were mainly of small sample size and thus may not have developed sufficient statistical power in their analyses (McIntyre and Jerrell, 2008; Burcu *et al*., 2018).

The risk of antipsychotic-associated cardiovascular and metabolic events may differ among countries (Man *et al*., 2020). This could potentially be due to variations in healthcare systems and preferences of patients and clinicians in the choice of antipsychotics (Pillinger *et al*., 2020). From the genetic perspective, the variant HTR2C gene, which encodes the 5HT2c receptor, may increase metabolic risk, especially by the rs 1414334 C allele (Ma *et al*., 2014). A survey indicated that this allele is present in a higher proportion of Americans (10%) and Europeans (15%), but, by contrast, is of very low frequency in Asians (1%). This suggests that variations between different ethnicities could affect the risk of cardiovascular and metabolic events (Mulder *et al*., 2009). To date, only limited evidence has been available comparing the corresponding risks among children and young adults receiving antipsychotics between different populations in real-world situations. We, therefore, conducted the current study with four large population-based datasets from Taiwan, Korea, Hong Kong and the UK, which have coverage of approximately 100 million individuals in total, to evaluate and benchmark the risk of specific cardiovascular events (stroke, IHD and AMI) and metabolic events (hypertension, T2DM and dyslipidaemia), associated with antipsychotics in children and young adults.

## Method

### Database sources

We included databases from Taiwan (Taiwan's National Health Insurance Database; NHID), Korea (Korea's NHID), Hong Kong (Clinical Data Analysis and Reporting System; CDARS) and the UK (The Health Improvement Network; THIN) in this study (Hsieh *et al*., 2019; Ilomäki *et al*., 2020). Additional details about the included databases are presented in Supplementary Table 1. We applied a distributed network approach with a common data model (CDM) (Lai *et al*., 2015). Briefly, the coordination centre distributed the common SAS program for analysis, generating aggregated results based on the CDM that standardised the structures and contents of participating databases, and collected the final summary of results from each participating site (Lai *et al*., 2015). This approach preserved the confidentiality of the data because the raw data stayed in the local site while the analyses were executed respectively by the sites (Lai *et al*., 2018*a*). Moreover, we could maintain the consistency of analysis among the sites through the use of the common analysis program (Lai *et al*., 2018*b*). The details of the mapping codes for diagnosis and the CDM are presented in Supplementary Table 2 and Supplementary Table 3. The study has been approved by the Human Research Ethics Committee at National Cheng Kung University (No. NCKU HREC-E-105-259-2); the University of Hong Kong/Hospital Authority Hong Kong West Cluster (No. UW15-619); The Health Improvement Network Scientific Review Committee (19THIN087) and the Institutional Review Board of Sungkyunkwan University (SKKU 2018-04-106).

### Study design

We applied a self-controlled case series (SCCS) design to investigate the association between antipsychotics and risk of metabolic/cardiovascular event, whereby the relative risk is estimated based on within-person comparisons rather than between-person comparisons (Hallas and Pottegård, 2014; Petersen *et al*., 2016). As a result, both measured and unmeasured time-independent confounding factors, such as sex, ethnicity, environmental and cultural factors are eliminated. The design is especially important for multinational studies, benchmarking results from different countries with very heterogeneous healthcare conditions (Lai *et al*., 2018*a*).

### Source population and exposure

We included patients aged 6–30 years diagnosed with a mental disorder (ICD-9-CM: 290–319) who were newly receiving oral antipsychotic drugs between 2004 and 2012 in Taiwan, 2010 and 2016 in Korea, 2001 and 2014 in Hong Kong and 1997 and 2016 in the UK. Incident use of antipsychotics was captured based on a 1-year washout period before the first record of antipsychotic prescription in the database. We excluded patients who had a record of congenital disorders, including congenital heart disease, familial hypercholesterolaemia and type 1 diabetes. We also excluded patients who had a cancer diagnosis record. Details of diagnostic codes are presented in Supplementary Table 3.

### Case identification and ascertainments

We included patients who had a record of the outcome of interest (cardiovascular events included AMI, IHD and stroke, and metabolic events included hypertension, dyslipidaemia and T2DM) for the analyses. To improve the validity of diagnoses of metabolic events, we confirmed cases by the records for corresponding drug prescriptions, including oral hypoglycaemic agents (A10B) for T2DM, lipid-modifying agents (C10) for dyslipidaemia and antihypertensive drugs including diuretics (C03A and C03B), beta-blockers (C07, except propranolol; C07AA05), calcium channel blockers (C08CA) and angiotensin-converting enzyme inhibitors (ACEI) / angiotensin receptor blockers (ARB) (C09).

### Definition of risk periods

**Fig. 1.**
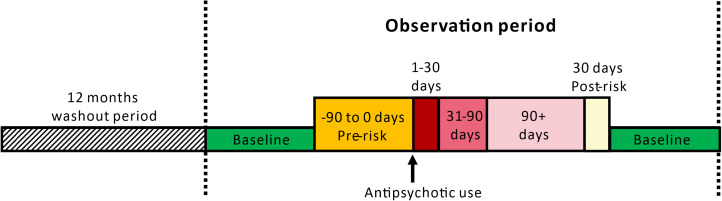
Schematic presentation of self-control case series.

We defined the observation period based on the availability of data sources as mentioned in the previous section. Observational periods began on the first available date in the corresponding database, or the sixth birthday of the patient (whichever was later) and ended on the last available date in the corresponding database, the 31^st^ birthday of the patient, or registered date of death (whichever was earlier). For each included participant, we defined the risk periods with respect to the duration of exposure to antipsychotics and categorised them into five mutually exclusive windows: (1) 90 days before (pre-exposure), (2) 1–30 days, (3) 31–90 days, (4) more than 90 days of antipsychotics use and (5) 30-day after the end of antipsychotics use (post-exposure) (Fig. 1). Details of ATC codes for antipsychotics are presented in Supplementary Table 4. A pre-exposure period was added to take account of the possibility that the outcome of interest may affect the likelihood of antipsychotics treatment, which in turn may introduce bias into the risk estimate during treatment whereas the post-exposure period acts as a washout period. To manage possible confounding effects due to age, we performed 1-year age banding for all patients in the analysis (Petersen *et al*., 2016).

### Statistical analysis

We report characteristics of patients included in the analyses by countries and describe categorical variables (e.g., sex) by number with proportion and continuous variables (e.g., age) by mean with standard deviation (SD). We considered the entire study period and categorised the risk periods in a time-varying manner, i.e. the exposure status was updated according to the treatment time. We calculate incidence rate ratio (IRR) and 95% confidence interval (95% CI) by conditional Poisson regression, adjusted for age in 1-year age bands to evaluate the risk of specific cardiovascular and metabolic events associated with antipsychotics in the different risk windows (Petersen *et al*., 2016). IRRs for each risk window in each site were pooled by the random-effect model (DerSimonian and Laird, 1986). The Taiwanese and Korean databases provided a sufficient number of cases to conduct a secondary analysis for the risk comparisons between individual antipsychotics.

## Results

### Antipsychotic users from each country

**Fig. 2.**
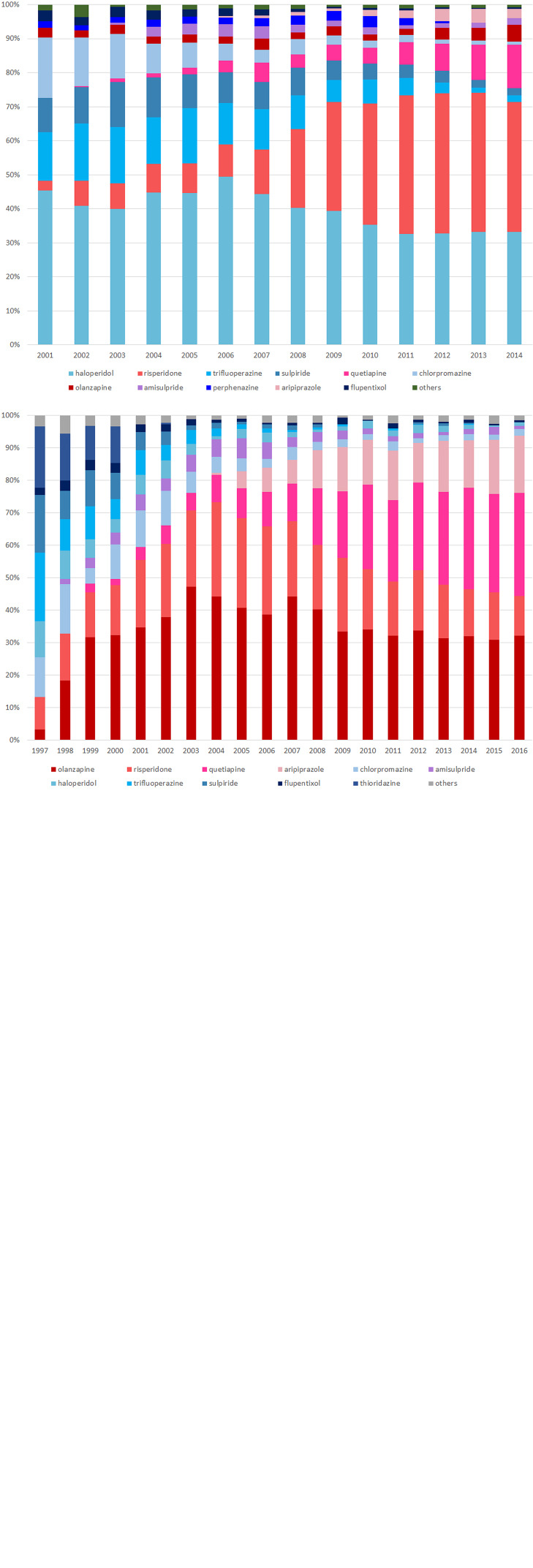
Utilisation pattern of antipsychotics among countries: (a) Taiwan (b) Korea (c) Hong Kong (d) UK.

We identified a total of 107 425 patients from Taiwan, 284 843 from Korea, 19 034 patients from Hong Kong and 7770 patients from the UK. The mean age (±SD) upon receiving the first prescription was 21.1 ± 6.7 years in Taiwan, 23.3 ± 5.3 years in Hong Kong and 24.9 ± 3.8 years in the UK. We found more males than females in Taiwan (61%), Korea (60%) and the UK (64%) and about the same males and females in Hong Kong (Table 1). The patterns and rates of antipsychotics use are presented in Fig. 2. The proportion of antipsychotics use varied among countries. The most commonly prescribed drugs at initiation were sulpiride in Taiwan, risperidone in Korea, haloperidol in Hong Kong and olanzapine in the UK.

**Table 1.** Baseline characteristics of all antipsychotic users included in the analysis

	Taiwan	Korea	Hong Kong	The UK
Number of patients	107 425	284 843	19 034	7770
Sex, *n* (%)				
Male	65 532 (61.0)	169 856 (59.6)	9711 (51.0)	4956 (63.8)
Female	41 893 (39.0)	114 987 (40.4)	9323 (49.0)	2814 (36.2)
Age (mean ± SD)	21.1 (±6.7)	19.9 (±6.9)	23.3 (±5.3)	24.9 (±3.8)
Age subgroup, *n* (%)				
6–12 years	16 091 (15.0)	50 829 (17.8)	667 (3.50)	7 (0.1)
13–18 years	18 986 (17.7)	67 759 (23.8)	3048 (16.0)	355 (4.6)
19–24 years	32 837 (30.6)	80 726 (28.3)	6451 (33.9)	3180 (40.9)
25–30 years	39 511 (36.8)	85 529 (30.3)	8868 (46.6)	4228 (54.4)

### Pooled analysis

**Fig. 3.**
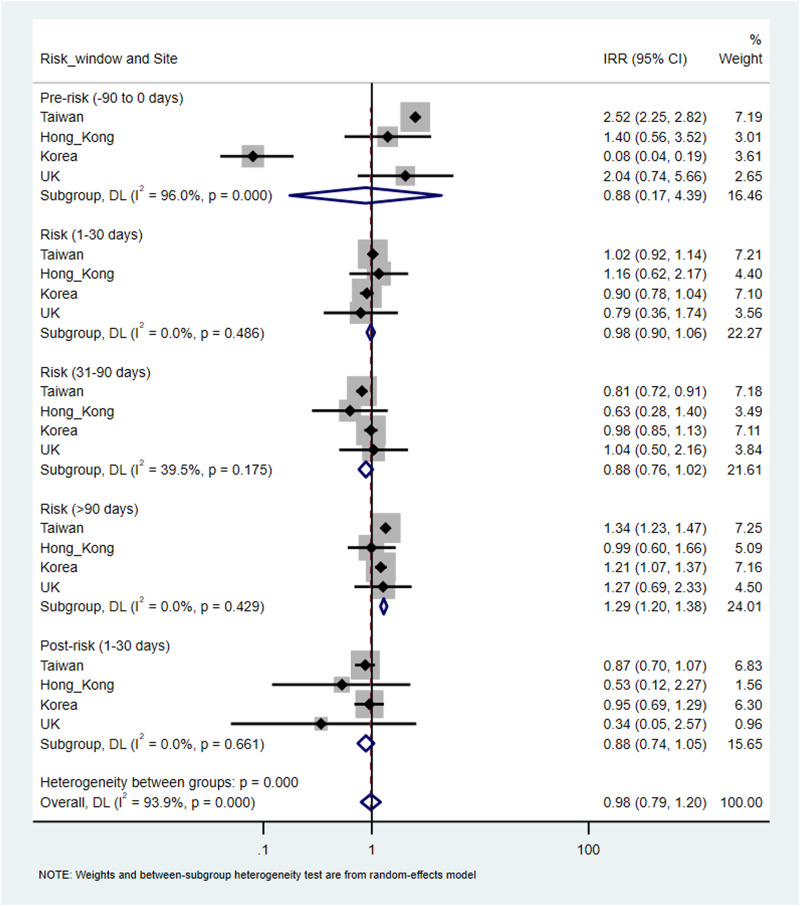
Pooled estimates of risk in metabolic events.

**Fig. 4.**
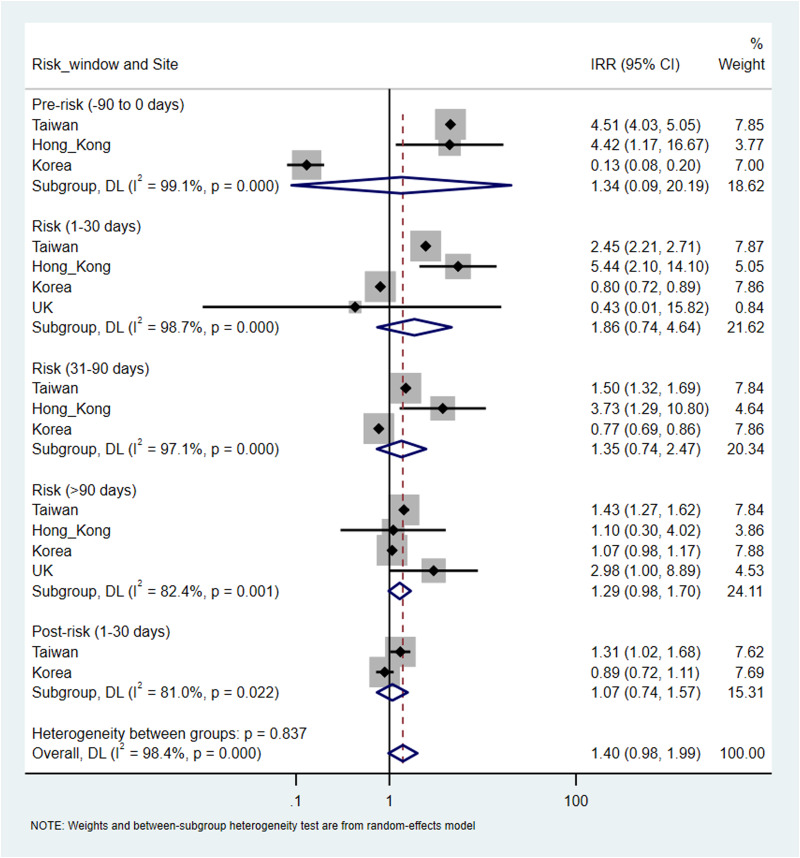
Pooled estimates of risk in cardiovascular events.

There were a total of 9730 (overall incidence rate, 11.5 per 100 person-years), 38 432 (12.4 per 100 person-years), 233 (7.6 per 100 person-years) and 120 (11.9 per 100 person-years) patients with at least one record of cardiovascular or metabolic events in Taiwan, Korea, Hong Kong and the UK, respectively. An increased risk of metabolic events was observed with more than 90-days of antipsychotics exposure with the pooled IRR of 1.29 (95% CI 1.20–1.38), but not in other risk windows. The corresponding IRR was 0.98 (95% CI 0.90–1.06) for 1–30 days and 0.88 (0.76–1.02) for 31–90 days of antipsychotics exposure. The I^2^ for heterogeneity ranged from 0% to 39.5% in the pooled estimates from metabolic events. For cardiovascular events, no significant association was identified in any risk windows in the pooled analysis. The pooled IRR was 1.86 (0.74–4.64) for 1–30 days, 1.35 (0.74–2.47) for 31–90 days and 1.29 (0.98–1.70) for more than 90-days of antipsychotics exposure, however, with high heterogeneity (*I*^2^ ranged from 82.4% to 98.7%). No significant associations were found in the pre- and post-exposure windows for both outcomes (Figs 3 and 4).

### Analysis by sites and specific events

The results of SCCS from individual sites varied. We found the risks of metabolic events were higher in the risk period of 90 + days in Taiwan and Korea. The IRR were 1.27 (1.12–1.45) and 1.48 (1.38–1.59) for hypertension, 1.35 (1.12–1.64) and 1.38 (1.28–1.49) for T2DM and 1.36 (1.20–1.55) and 1.36 (1.28–1.49) for dyslipidaemia in Taiwan and Korea, respectively. However, the risk of metabolic events in the period of 90 + days was higher but not reached statistical significance in Hong Kong and the UK (Table 2).

**Table 2.** Risk of metabolic events among countries

	Taiwan NHID				Korea NHID				Hong Kong CDARS				UK THIN			
	Events	Person-years	IRR	(95% CI)	Events	Person-years	IRR	(95% CI)	Events	Person-years	IRR	(95% CI)	Events	Person-years	IRR	(95% CI)
Metabolic risk																
Baseline	3898	37 852	1.00	(reference)	20 495	163 759	1.00	(reference)	89	1581	1.00	(reference)	44	511	1.00	(reference)
Pre-risk (−90 to 0 days)	352	1207	2.52	(2.25–2.82)	1763	23 396	0.08	(0.04–0.19)	5	40	1.40	(0.56–3.52)	6	19	2.04	(0.74–5.66)
Risk (1–30 days)	437	3935	1.02	(0.92–1.14)	1980	23 231	0.90	(0.78–1.04)	13	137	1.16	(0.62–2.17)	10	93	0.79	(0.36–1.74)
Risk (31–90 days)	375	3891	0.81	(0.72–0.91)	9333	54 134	0.98	(0.85–1.13)	7	136	0.63	(0.28–1.40)	13	92	1.04	(0.50–2.16)
Risk (90 + days)	1130	6825	1.34	(1.23–1.47)	61	5486	1.21	(1.07–1.37)	74	619	0.99	(0.60–1.66)	42	240	1.27	(0.69–2.33)
Post-risk (1–30 days)	90	838	0.87	(0.70–1.07)	447	3953	0.95	(0.69–1.29)	2	23	0.53	(0.12–2.27)	1	16	0.34	(0.05–2.57)
Risk of hypertension																
Baseline	2038	19 906	1.00	(reference)	4886	39 465	1.00	(reference)	45	726	1.00	(reference)	9	92	1.00	(reference)
Pre-risk (−90 to 0 days)	201	623	2.81	(2.42–3.26)	11	1355	0.06	(0.03–0.11)	2	18	0.89	(0.21–3.75)	3	4	7.72	(1.40–42.68)
Risk (1–30 days)	251	1996	1.16	(1.01–1.34)	480	6172	0.61	(0.56–0.68)	9	55	1.41	(0.64–3.11)	1	16	0.57	(0.06–5.14)
Risk (31–90 days)	209	1974	0.90	(0.77–1.05)	587	6130	0.72	(0.65–0.79)	3	55	0.47	(0.14–1.59)	2	16	1.17	(0.21–6.60)
Risk (90 + days)	530	3252	1.27	(1.12–1.45)	2667	14 861	1.48	(1.38–1.59)	23	208	0.55	(0.24–1.26)	9	38	2.18	(0.53–9.03)
Post-risk (1–30 days)	38	427	0.72	(0.52–1.00)	118	1015	0.88	(0.73–1.06)	1	10	0.41	(0.05–3.24)	0	3	N/A	(---- ----)
Risk of type 2 DM																
Baseline	791	7618	1.00	(reference)	4154	33 428	1.00	(reference)	37	672	1.00	(reference)	19	208	1.00	(reference)
Pre-risk (−90 to 0 days)	64	254	2.13	(1.64–2.77)	11	1158	0.07	(0.04–0.13)	2	18	1.52	(0.35–6.65)	2	8	1.34	(0.24–7.37)
Risk (1–30 days)	85	867	0.88	(0.69–1.11)	422	5562	0.62	(0.56–0.69)	3	73	0.57	(0.17–1.96)	5	40	0.94	(0.29–3.07)
Risk (31–90 days)	78	857	0.79	(0.61–1.01)	389	5525	0.54	(0.48–0.60)	3	73	0.57	(0.17–1.97)	7	40	1.34	(0.45–3.98)
Risk (90 + days)	286	1629	1.35	(1.12–1.64)	2441	13 957	1.38	(1.28–1.49)	43	338	1.34	(0.65–2.76)	17	101	1.08	(0.41–2.86)
Post-risk (1–30 days)	22	184	0.86	(0.56–1.33)	106	887	0.84	(0.69–1.03)	0	12	N/A		0	7	N/A	(---- ----)
Risk of dyslipidaemia																
Baseline	1773	17 368	1.00	(reference)	14 562	116 747	1.00	(reference)	12	316	1.00	(reference)	18	230	1.00	(reference)
Pre-risk (−90 to 0 days)	139	563	2.19	(1.83–2.61)	45	3885	0.09	(0.06–0.12)	1	7	2.33	(0.27–20.11)	1	8	0.92	(0.08–10.32)
Risk (1–30 days)	163	1908	0.78	(0.66–0.92)	1123	16 207	0.56	(0.52–0.59)	1	27	0.81	(0.10–6.87)	4	40	0.82	(0.23–2.94)
Risk (31–90 days)	165	1887	0.71	(0.60–0.85)	1292	16 091	0.64	(0.61–0.68)	1	27	0.82	(0.10–6.90)	4	39	0.82	(0.23–2.95)
Risk (90 + days)	613	3534	1.36	(1.20–1.55)	6376	37 150	1.38	(1.34–1.42)	21	160	2.59	(0.77–8.67)	19	110	1.43	(0.56–3.63)
Post-risk (1–30 days)	52	399	0.98	(0.74–1.30)	303	2771	0.88	(0.78–1.00)	1	4	2.69	(0.25–29.41)	1	6	0.81	(0.10–6.73)

NHID, National Health Insurance Database; CDARS, Clinical Data Analysis and Reporting System, THIN, The Health Improvement Network, DM, diabetes mellitus, N/A, not available.

Compared to the non-exposure period, we found the risk of stroke to be higher in the pre-exposure period (5.49; 4.72–6.38), 1–30 days (3.51; 3.07–4.01), 31–90 days (2.06; 1.75–2.42) and 90 + days (2.07; 1.77–2.43) and the risk of IHD (1.59; 1.35–1.86) and AMI was higher in the pre-exposure period (6.42; 2.86–14.45) and the risk period of 1–30 days (2.29; 0.91–5.78) in Taiwan (Table 3). We did not find an association between antipsychotics and cardiovascular events in Korea, except for a higher risk of stroke in the period of 90 + days (1.38; 1.34–1.41) comparing with the non-exposure period. In Hong Kong, we found the risk of cardiovascular events, specifically for stroke (5.80; 1.91–17.61) was higher in the risk period of 1–30 days compared to the non-exposure period. We did not find any association between the use of antipsychotics and the risk of cardiovascular events in the UK.

**Table 3.** Risk of cardiovascular events among countries

	Taiwan NHID				Korea NHID				Hong Kong CDARS				UK THIN			
	Events	Person-years	IRR	(95% CI)	Events	Person-years	IRR	(95% CI)	Events	Person-years	IRR	(95% CI)	Events	Person-years	IRR	(95% CI)
Risk of cardiovascular events																
Baseline	2480	27 250	1.00	(reference)	4209	32 891	1.00	(reference)	17	402	1.00	(reference)	4	34	1.00	(reference)
Pre-risk (−90 to 0 days)	367	815	4.51	(4.03–5.05)	19	1101	0.13	(0.08−0.20)	3	9	4.42	(1.17–16.67)	0	1	N/A	(---- ----)
Risk (1–30 days)	506	2384	2.45	(2.21–2.71)	443	4159	0.80	(0.72−0.89)	10	31	5.44	(2.10–14.10)	1	4	0.43	(0.01-15.82)
Risk (31–90 days)	324	2357	1.50	(1.32–1.69)	456	4128	0.77	(0.69−0.86)	7	31	3.73	(1.29–10.80)	0	4	N/A	(---- ----)
Risk (90 + days)	467	3386	1.43	(1.27–1.62)	1231	8537	1.07	(0.98−1.17)	8	85	1.10	(0.30–4.02)	1	12	12	(0.00-8.89)
Post-risk (1–30 days)	65	531	1.31	(1.02–1.68)	86	749	0.89	(0.72−1.11)	0	6	N/A	(---- ----)	0	1	N/A	(---- ----)
Risk of stroke																
Baseline	1175	14 308	1.00	(reference)	2042	16 312	1.00	(reference)	12	293	1.00	(reference)	4	31	1.00	(reference)
Pre-risk (−90 to 0 days)	212	434	5.49	(4.72–6.38)	6	547	0.09	(0.07–0.11)	2	7	4.29	(0.87–21.23)	0	1	N/A	(---- ----)
Risk (1–30 days)	321	1253	3.51	(3.07–4.01)	226	2055	0.60	(0.57–0.63)	8	25	5.80	(1.91–17.61)	0	3	N/A	(---- ----)
Risk (31–90 days)	196	1239	2.06	(1.75–2.42)	267	2040	0.68	(0.65–0.71)	6	24	4.25	(1.26–14.26)	0	3	N/A	(---- ----)
Risk (90 + days)	284	1779	2.07	(1.77–2.43)	636	4310	1.38	(1.34–1.41)	6	69	1.01	(0.21–4.76)	1	10	0.51	(0.01–20.82)
Post-risk (1–30 days)	33	276	1.50	(1.06–2.14)	41	366	0.90	(0.82–0.99)	0	5	N/A	(---- ----)	0	1	N/A	(---- ----)
Risk of IHD																
Baseline	1353	13 388	1.00	(reference)	2158	16 533	1.00	(reference)	3	75	1.00	(reference)	0	2	1.00	(reference)
Pre-risk (−90 to 0 days)	155	393	3.51	(2.96–4.17)	13	551	0.17	(0.10–0.29)	1	2	13.34	(0.42–428.54)	0	0.2	N/A	(---- ----)
Risk (1–30 days)	196	1182	1.59	(1.35–1.86)	209	2079	0.71	(0.61–0.82)	1	5	9.42	(0.34–258.63)	1	0.3	N/A	(---- ----)
Risk (31–90 days)	125	1169	0.95	(0.77–1.15)	196	2063	0.62	(0.52– 0.72)	1	5	9.42	(0.34–258.63)	0	0.3	N/A	(---- ----)
Risk (90 + days)	196	1675	0.95	(0.79–1.14)	576	4151	0.94	(0.83–1.07)	1	14	N/A	(---- ----)	0	0.3	N/A	(---- ----)
Post-risk (1–30 days)	32	265	1.07	(0.75–1.53)	47	380	0.87	(0.65–1.17)	0	1	N/A	(---- ----)	0	0.1	N/A	(---- ----)
Risk of AMI																
Baseline	35	372	1.00	(reference)	122	990	1.00	(reference)	2	34	1.00	(reference)	0	2	1.00	(reference)
Pre-risk (−90 to 0 days)	8	12	6.42	(2.86–14.45)	0	34	N/A	(---- ----)	0	0.2	N/A	(---- ----)	0	0.2	N/A	(---- ----)
Risk (1–30 days)	6	34	2.29	(0.91–5.78)	19	127	1.22	(0.71–2.10)	1	2	N/A	(---- ----)	0	0.4	N/A	(---- ----)
Risk (31–90 days)	1	33	0.39	(0.05–2.92)	13	127	0.88	(0.48–1.63)	0	2	N/A	(---- ----)	0	0.4	N/A	(---- ----)
Risk (90 + days)	6	42	1.05	(0.31–3.54)	43	278	1.37	(0.84–2.24)	1	2	N/A	(---- ----)	0	0.9	N/A	(---- ----)
Post-risk (1–30 days)	0	8	N/A	(---- ----)	1	23	0.46	(0.06–3.31)	0	0.4	N/A	(---- ----)	0	0.1	N/A	(---- ----)

NHID, National Health Insurance Database; CDARS, Clinical Data Analysis and Reporting System; THIN, The Health Improvement Network; IHD, ischaemic heart disease, AMI, acute myocardial infarction; N/A, not available.

## Discussion

Previous studies evaluating the safety of antipsychotics in young people were generally with a limited sample size to acquire a precise estimation (McIntyre and Jerrell, 2008; Burcu *et al*., 2018). The current study used four large databases from Taiwan, Korea, Hong Kong and the UK to evaluate the risk of cardiovascular and metabolic events associated with antipsychotics in children, adolescents and young adults. We identified an increased risk of metabolic events with exposure to antipsychotics for more than 90 days from the pooled results. However, no significant association was found between the use of antipsychotics and the risk of cardiovascular events. This suggested that the increased risk in the metabolic events may not be severe enough to trigger cardiovascular events in children, adolescents and young adults. Despite the varied risk pattern among countries, the conclusion is consistent with the overall results.

The results of our study were largely consistent with previous studies regarding the association between the risk of metabolic events with antipsychotics use (Man *et al*., 2020). However, we found an increased risk of metabolic events only with the antipsychotics exposure for more than 90 days, implicating it may require a period of accumulative exposure of drugs to develop metabolic events. Our pooled analysis did not support the use of antipsychotics was associated with cardiovascular events. However, the finding was based on the results from countries with high heterogeneity. We found an increased risk of cardiovascular events in Taiwan and a specifically higher risk of stroke in Hong Kong in the first 30 days of antipsychotics treatment. In particular, we found patients receiving haloperidol had an increased risk of stroke in the initial stage of treatment in both Taiwan and Hong Kong. Haloperidol has a high affinity for binding to *α*1 and *α*2 receptors (Hensiek and Trimble, 2002), and the blockade of *α* receptors could cause fluctuations in blood pressure along with some symptoms such as hypotension, hypertension and QT interval prolongation, leading to a high risk of cardiovascular events (Cooper *et al*., 2016; Hiremath *et al*., 2019). However, a more parsimonious interpretation of this pattern of temporal association is that the observed increased risk of cardiovascular events is not due to antipsychotics but precedes it because an increased risk was also observed in 90 days pre-exposure period from both Taiwan and Hong Kong. The changes in behavioural and mental health symptoms or associated impairment that lead to a medical consultation or comorbidities, which in turn may contribute to the decision to prescribe antipsychotics.

Besides, it is noteworthy that different risk profiles among countries could be attributed to different patterns of antipsychotic or prescribing preferences among the countries. From the study cohorts, we found that SGA uses increased over the years in Korea and the UK, and until 2016, SGA accounted for more than 80% of total antipsychotics use in children, adolescents and young adults. The frequent use of SGA may explain the higher risk of metabolic events within the observation window of more than 90 days after drug initiation, compared to the non-exposure period. We suggest interpreting the results cautiously considering countries’ specific situations. For instance, the healthcare accessibility or copayment of medical treatment may be different among countries, leading to various thresholds for seeking medical treatment (Lai *et al*., 2018*b*). The possibilities of the capture of events were different among countries. Moreover, the differences in the respective healthcare systems, cultures, behaviours of prescribing, preferences of clinicians among countries may also contribute to the heterogeneity of the results. Therefore we could not make inferences on the ethnic differences regarding the adverse effects in our study.

### Limitations

We were unable to assess the actual medication adherence of patients, which may cause a bias towards null because patients may or may not be taking the medication. The self-controlled design eliminated time-constant unmeasured confounders, but the results may be influenced by time-variant factors that were not associated with age (Hallas and Pottegård, 2014; Petersen *et al*., 2016). Protopathic bias should be noted with patients who had undetected cardiovascular events that caused psychosis and the use of antipsychotics. Because clinicians may avoid antipsychotics which are well known to increase metabolic side effects, such as olanzapine for patients with higher baseline risk, the sample size and power of the study may be decreased. On the other hand, because metabolic monitoring is not being implemented routinely in all study countries, we may omit some patients with mild metabolic syndrome.

## Conclusion

Exposure to antipsychotics for more than 90 days was associated with an increased risk of a metabolic event, but did not trigger cardiovascular events in children and young adults. Although we found varied risk profiles of cardiovascular and metabolic events between countries, the conclusion remained consistent with the overall results. Nevertheless, clinicians should be mindful of the possible cardiovascular and metabolic risk as with the use of all antipsychotics in children and young adults in clinical practice while long-term use of antipsychotics is required.

## Supplementary material

For supplementary material accompanying this paper visit http://dx.doi.org/10.1017/S2045796021000494. click here to view supplementary material

## Data

All data are not publicly available due to the issue of privacy. Requests for data require the review by the custodian from each country.
