# Supplementary material for: Cardiovascular and metabolic risk of antipsychotics in children and young adults: a multinational self-controlled case series study
Source: Epidemiol Psychiatr Sci. 2021 Oct 15;30:e65. doi: 10.1017/S2045796021000494 (PMC8546502; doi:10.1017/S2045796021000494)
Supplement: Supplementary file 1 [file S2045796021000494sup001.docx]

| **Supplementary Table1.** Participating Data Source |
| --- |
| **Taiwan’s NHID (2004-2012)** |
| The National Health Insurance Research Database (NHIRD) in Taiwan is maintained and made accessible for research purposes by the National Health Research Institute (NHRI). Taiwan launched a single-payer, mandatory National Health Insurance program, and by 2011, the entire Taiwanese population had been enrolled. The NHRI compiles information on enrollees' demographics, health care professionals and facilities, service claims from inpatient and ambulatory care, and contracted pharmacies for reimbursement purposes. Personal identities are encrypted for privacy protection, but all data sets can be linked by unique, anonymous identifiers created by NHRI. All the antipsychotics and most prescription drugs are reimbursed by NHI in Taiwan, and all records of reimbursed drugs from inpatient, outpatient, emergency services and contracted pharmacy settings are included in NHIRD. Accuracy of the major disease diagnoses in the NHIRD, such as stroke, epilepsy, and acute coronary syndrome, has been validated. |
| **South Korea Health Insurance Database (2010-2016)** |
| The South Korean Health Insurance database has been widely used in the pharmaco-epidemiology field. Korea’s national health insurance program was initiated in 1977 and achieved universal coverage of the entire population by 1989. The database contains all information on diagnoses and prescribed drugs for about 50 million Koreans. The database includes an anonymized identifier representing each individual together with age, sex, diagnoses, and prescription drugs. Information on prescribed drugs includes generic name, prescription date, duration, and route of administration. In particular, the Korean database includes all prescription information both in outpatient and inpatient settings, owing to a specific fee-for-service system. All diagnoses are coded according to ICD-10. Previous validation studies have compared the diagnoses derived from the claims database with the ideal of actual diagnoses recorded in the patients’ medical records obtained from hospital or clinic chart review. The overall positive predictive value of all diagnoses was about 70%. |
| **Clinical Data Analysis and Reporting System of Hong Kong (2001-2014)** |
| The Hong Kong Hospital Authority is a statutory body that manages all public hospitals and their outpatient clinics in Hong Kong. The Hospital Authority not only provides acute hospital care, but also provides acute and chronic disease management to patients in the community via outpatient clinics (both specialists and general physicians) throughout Hong Kong. Health services are available to all Hong Kong residents (over 7 million people). Data were extracted from the Clinical Data Analysis and Reporting System (CDARS), a database developed by the Hospital Authority. In 1995, the Hospital Authority developed the Clinical Management System (CMS), an electronic health record system that allows clinicians to order, document, and review care in their daily practice. CMS contains patients’ data, including demographic information, diagnosis, payment method, prescription information, laboratory tests, and hospital admission and discharge information. Drug information is stored in the system with prescribing details (e.g., drug name, dose, drug frequency). Data from CMS are transferred to CDARS for research and audit purposes. CDARS also contains a multitude of data warehouses, including the Accident and Emergency Information System, Medical Record Abstract System, In-Patient Administration System, and the Pharmacy Management System/Corporate Drug Dispensing History. Patient records in CDARS are anonymous (patient names, Hong Kong identification card numbers, addresses, and telephone numbers are not available) to protect patient confidentiality. A unique patient reference number is generated for each individual case to facilitate data retrieval and further analysis. CDARS has captured data since 1995. |
| **The UK－THIN (1997-2016)** |
| Two main large-scale databases in the UK are Clinical Practice Research Datalink (CPRD) and The Health Improvement Network (THIN). CPRD includes medical records data from 684 practices, representing approximately 15 million UK patients, and THIN contains records from 587 practices, representing approximately 12 million UK patients. The THIN database was established in 2003 as a collaboration between In Practice Systems (INPS) who developed Vision software used by general practitioners (GPs) in the UK to manage patient data, and IMS Health who then provide access to the data for use in medical research. The UCL Research Departments Primary Care & Population Health (PCPH) and Infection & Public Health (IPH) have acquired a full license to THIN for the purposes of conducting large-scale epidemiological, clinical and health care utilization studies. The database contains records of 12 million patients, equivalent to 75.6 million patient years, covering 6.2% of the UK population. Patient files include sex, age and date of entering and leaving. Medical diagnoses are coded according to Read code and medications according to ATC code. All data are fully anonymous, processed and validated by CSD Medical Research UK. |

|  | | |
| --- | --- | --- |
| **Supplementary Table 2**. Diagnosis code in the study | | |
| Variables | ICD-9 | ICD-10 |
| Mental disorder | 290-319 | F00-F99 |
| Cancer | 140-239 | C00-D48 |
| Congenital heart disease | 745-747 | Q20-Q24 |
| Type 1 diabetes mellitus | 250.X1, 250.X3  (X=0-9) | E10 |
| Stroke | 430-437 | I60-69 |
| Ischemic heart disease (IHD) | 411, 413, 414 | I20-25 |
| Acute myocardial infarction (AMI) | 410 | I21 |
| Hypertension | 401-405 | I10-15 |
| Type 2 diabetes mellitus (DM) | 250.X0, 250.X2  (X=0-9) | E11-14 |
| Dyslipidemia | 272 | E78 |

**Supplementary Table 3**. Common data model

Demographic table

| Id | Id_birthday | Sex  (male=1, female=0, others=999) |
| --- | --- | --- |
| 10001 | 1972-09-08 | 1 |
| 10002 | 1990-12-28 | 0 |
| 10003 | 1993-11-05 | 999 |

Eligible table

| Id | Enrol_in_date | Enrol_out_date |
| --- | --- | --- |
| 10001 | 1995-03-01 | 2013-12-31 |
| 10002 | 1995-03-01 | 2012-09-08 |
| 10003 | 2000-10-08 | 2013-05-06 |
| 10004 | 2005-07-20 | 2013-12-31 |

Medication table

| Id | Atccode* | Rx_date | Rx_end | Supply_day | Setting  (IP=inpatient; OP=outpatient) |
| --- | --- | --- | --- | --- | --- |
| 10001 | N05AH03 | 2013-08-01 | 2013-08-07 | 7 | IP |
| 10001 | N05AH03 | 2013-09-01 | 2013-09-29 | 28 | IP |
| 10001 | A10BA02 | 2014-10-12 | 2014-10-18 | 7 | OP |
| 10001 | A10BA02 | 2015-10-05 | 2015-10-18 | 14 | IP |

Diagnostic table

| Id | Icd9* | Event_date | Setting  (IP=inpatient; OP=outpatient) |
| --- | --- | --- | --- |
| 10001 | 4010 | 2013-09-01 | IP |
| 10002 | 41301 | 2014-10-08 | IP |
| 10003 | 431 | 2015-07-20 | OP |
| 10004 | 25002 | 2015-09-30 | OP |

*Icd9=ICD-9 (NHIRD), ICD-10 (CDARS), Read code (THIN)

**Supplementary Table 4**. **ATC codes for antipsychotics**

| ATC code | Drug |
| --- | --- |
| First-generation antipsychotics | |
| N05AA01 | Chlorpromazine |
| N05AA02 | Levomepromazine |
| N05AA05 | Triflupromazine |
| N05AB02 | Fluphenazine |
| N05AB03 | Perphenazine |
| N05AB06 | Trifluoperazine |
| N05AC02 | Thioridazine |
| N05AD01 | Haloperidol |
| N05AD08 | Droperidol |
| N05AF01 | Flupentixol |
| N05AF02 | Clopenthixol |
| N05AF03 | Chlorprothixene |
| N05AF04 | Tiotixene |
| N05AF05 | Zuclopenthixol |
| N05AG02 | Pimozide |
| N05AL01 | Sulpiride |
| Second-generation antipsychotics | |
| ATC code | Drug |
| N05AE04 | Ziprasidone |
| N05AE05 | Lurasidone |
| N05AH01 | Loxapine |
| N05AH02 | Clozapine |
| N05AH03 | Olanzapine |
| N05AH04 | Quetiapine |
| N05AL05 | Amisulpride |
| N05AX08 | Risperidone |
| N05AX11 | Zotepine |
| N05AX12 | Aripiprazole |
| N05AX13 | Paliperidone |
| N05AX14 | Iloperidone |
